# Insights into the role of PGF2α in canine periparturient myometrium

**DOI:** 10.3389/fphys.2024.1392080

**Published:** 2024-05-28

**Authors:** Carolin Jungmann, Signe Dyhrberg Haubuff, Eva-Maria Packeiser, Hanna Körber, Iris Margaret Reichler, Orsolya Balogh, Gemma Mazzuoli-Weber, Sandra Goericke-Pesch

**Affiliations:** ^1^ Unit for Reproductive Medicine—Clinic for Small Animals, University for Veterinary Medicine Hannover, Foundation, Hannover, Germany; ^2^ Section for Veterinary Reproduction and Obstetrics, Department of Clinical Veterinary Sciences, University of Copenhagen, Taastrup, Denmark; ^3^ Clinic of Reproductive Medicine, University of Zurich Vetsuisse Faculty, Zurich, Switzerland; ^4^ Department of Small Animal Clinical Sciences, Virginia-Maryland College of Veterinary Medicine, Blacksburg, VA, United States; ^5^ Institute for Physiology and Cell Biology, University of Veterinary Medicine Hannover, Hannover, Germany

**Keywords:** canine, parturition, myometrium, prostaglandin F2α, uterine inertia

## Abstract

Parturition in dogs is subjected to complex hormonal regulation, with the involvement of prostaglandin F2α (PGF2α) still not fully understood. To investigate uterine inertia (UI), the most prevalent maternal reason for dystocia in the bitch, a better understanding of undisturbed uterine, especially myometrial function, is crucial. Our aim was to gain deeper insights into the role of PGF2α in the canine parturient myometrium. Uterine biopsies were obtained during medically indicated cesarean sections. To test for stimulatory effects of PGF2α *in vitro*, circular and longitudinal myometrial layer tissue strips were challenged with 50 pM, 0.5 µM, and 50 µM PGF2α. *Prostaglandin-endoperoxide synthase* 2 (*PTGS2*) and *PGF2α-receptor* (*PTGFR*) mRNA expressions were compared between primary UI (PUI) and obstructive dystocia (OD) samples in isolated parturient myometrium. PTGFR protein expression was assessed in full thickness uterine samples. PGF2α concentrations were analyzed in canine interplacental tissue around term. In the organ bath, the contractile response to PGF2α was limited to the circular layer at the highest dosage. Correspondingly, PTGFR immunohistochemical staining was significantly stronger in the circular layer (*p* ≤ 0.01). *PTGS2* gene expression did not differ between PUI and OD, whereas *PTGFR* gene expression could not be quantified. Local uterine PGF2α concentrations correlated negatively with serum P4 levels and were the highest during prepartum luteolysis while being significantly lower in PUI. Conclusively, despite the significant increase in local PGF2α concentrations at birth, confirming the interplacental tissue as a production site, our results suggest that PGF2α might affect uterine contractility during labor, mainly indirectly.

## 1 Introduction

Parturition in dogs involves very complex hormonal regulation. However, the underlying prepartal regulatory mechanisms leading to the initiation of canine labor have been poorly understood so far. Canine pregnancy is maintained by progesterone (P4) of exclusively luteal origin, as the placenta lacks steroidogenic activity (Hoffmann et al., 1994; Nishiyama et al., 1999). The onset of parturition is preceded by a dramatic drop in P4 12–24 h before the first clinical signs of labor become visible (Concannon et al., 1977; Concannon et al., 1989; Veronesi et al., 2002). Simultaneously, a rapid increase in circulating prostaglandin F2a (PGF2α), measurable as its metabolite, 13,14-dihydro-15-keto-prostaglandin F2-alpha (PGFM), occurs (Concannon et al., 1977; Concannon et al., 1988; Nohr et al., 1993; Baan et al., 2005; Kowalewski et al., 2010; Gram et al., 2013; Kowalewski, 2014). Thus, different from the slow luteal regression in non-pregnant bitches, which is apparently biologically preprogrammed and associated with luteal aging and degeneration (Zatta et al., 2017), prepartum luteolysis is an actively regulated process in the dog (Kowalewski, 2014; Kowalewski et al., 2015a). The prepartum increase in PGF2α in bitches is suggested to originate from the placental trophoblast cells, as upregulation of prostaglandin-endoperoxide synthase 2 (PTGS2) in the fetal trophoblast was observed in response to altered feto-maternal communication (Kowalewski et al., 2010). Increased PTGS2 indicates the activation of the prostaglandin system. The reduced activity of the specific PGF2α-synthase (PGFS/AKR1C3), concomitant with increased expression of the PGE2-synthase (PTGES), demonstrates that an alternative pathway with PGE2 as substrate is crucial for local prepartum PGF2α synthesis (Kowalewski et al., 2010; Gram et al., 2013; Gram et al., 2014). Additionally, *in vitro*, it was shown that the endometrium of pregnant bitches produces PGF2α immediately prepartum (Luz et al., 2006). Following luteolysis, the P4-mediated inhibition of myometrial contractions in the pregnant canine uterus ceases, and the uterus becomes sensitive to contractile stimuli (Concannon et al., 1989; Lyse and Challis, 1989; Verstegen-Onclin and Verstegen, 2008). Myometrial contractile activity at parturition is generated by increased levels of contraction-associated proteins, including actin, myosin, and gap junction proteins, especially connexin 43 and the oxytocin receptor, but also PTGS2 and the receptors for PGF2α and PGE2 (PTGFR and PTGER1-4) (Cook et al., 2000; Patel and Challis, 2001; Challis et al., 2002; Olson, 2003; Kowalewski et al., 2010; Gram et al., 2013; Gram et al., 2014). Uterine contractions can then be evoked by contractile stimulators such as oxytocin and prostaglandins (Challis et al., 2002). Nevertheless, detailed knowledge of these processes in dogs is missing. A good understanding of myometrial (patho)physiology is crucial, especially in relation to the most common birth-associated pathology in bitches, uterine inertia (UI). UI is responsible for up to 70% of maternally caused dystocia cases, and in the absence of successful medical treatment options, a cesarean section is often performed immediately after diagnosis (Darvelid and Linde-Forsberg, 1994; Bergström et al., 2006; Prashantkumar and Walikar, 2018). However, in secondary uterine inertia (SUI), exhaustion of the myometrium occurs, usually because of prolonged labor due to a specific cause of dystocia, such as obstruction. The reasons for myometrial contractile failure in primary uterine inertia (PUI) are not fully elucidated (Feldman and Nelson, 2004; Linde Forsberg and Eneroth, 2005). Predisposing factors such as obesity, extremely small or large litter size, increasing age, nutritional and hormonal imbalances, stress, systemic diseases, and hypocalcemia or hypoglycemia of the bitch have been postulated (Johnston, 1986; Darvelid and Linde-Forsberg, 1994; Linde Forsberg and Persson, 2007; Münnich and Küchenmeister, 2009; Davidson, 2011). At the mRNA level, smooth muscle γ-actin, smooth muscle myosin heavy chain 11, and the Rho-associated coiled-coil-containing protein kinases 1 and 2 (involved in calcium sensitization and muscle contractions) are differentially expressed in the uterus between PUI and obstructive dystocia (OD) (Egloff et al., 2020; Frehner et al., 2021). In contrast, the expression of leptin as well as serum-ionized calcium and glucose concentrations did not differ between PUI and OD (Frehner et al., 2018; Frehner et al., 2021). Recently, compared to bitches with a fetal cause for dystocia, downregulated relative gene expression of myosin light chain kinase 4 was found in complete and partial PUI (Magnus et al., 2023). Additionally, in partial PUI, smooth muscle myosin heavy chain 2 was downregulated and protein kinase C expression increased, suggesting the possibility of a defect at the myometrial level (Magnus et al., 2023).

Although the failure of luteolysis was postulated to be causative for UI (van der Weyden et al., 1989; Irons et al., 1997; Bergström et al., 2010; McLean, 2012), it could not be confirmed in a recent study of our group (Jungmann et al., 2022). However, *in vivo* and *in vitro* studies showed a contractile response of the myometrium to exogenous PGF2α in humans (Senior et al., 1992; Senior et al., 1993; Patel and Challis, 2001; Janicek et al., 2007; Fischer et al., 2008), cattle (Takagi et al., 2002), and rats (Balki et al., 2010; Balki et al., 2012). The importance of prostaglandins as uterotonic agents has been postulated in dogs (Nohr et al., 1993; Hoffmann et al., 1999), but investigations of PGF2α′s actions in the canine myometrium are scarce. Gogny et al. (2010) assessed the uterokinetic properties of PGF2α on uterine tissue from cyclic bitches *in vitro*, and Ibuki et al. (1997) implanted force transducers in the uteri of non-pregnant bitches, measuring PGF2α-induced contractions. Regarding parturition, functional studies investigating the effect of PGF2α on canine pregnant myometrium are completely lacking. Similarly, local PGF2α concentrations were determined in canine corpora lutea (Kowalewski et al., 2015b), but the actual PGF2α concentration in the uterus itself at term is unknown. Previous studies investigating the prostaglandin system in relation to UI in dogs failed to identify significant correlations in uterine full-thickness biopsies (Rempel et al., 2021a; Rempel et al., 2021b). However, since oxytocin receptor expression and response to oxytocin stimulation *in vitro* differ between the two myometrial layers (Jungmann et al., 2022; Jungmann et al., 2023), slightly different functions of the circular and longitudinal myometrial layers can be assumed. To test for the hypothesized uterokinetic effect of PGF2α, we used exogenous PGF2α and investigated its ability to initiate canine myometrial contractions *in vitro*. Further aims of our study were to determine local PGF2α concentrations in interplacental tissue samples from bitches around parturition and investigate the mRNA and protein expression of PTGS2 and PTGFR in parturient canine uterine tissues in the context of PUI, considering the expression in the different myometrial layers.

## 2 Materials and methods

### 2.1 Animals and grouping

Animal experimentation was approved by the respective authorities (Lower Saxony State Office for Consumer Protection and Food Safety (LAVES), permit AZ 20/3360; Cantonal Veterinary Office Zurich, permit no. ZH086/15; Animal Experiments Inspectorate, Danish Veterinary and Food Administration, Ministry of Food, Agriculture, and Fisheries of Denmark, permit no. 2015-15-0201-00513). For all experiments, uterine tissue from bitches at term was used. Bitches included in this study were presented for medically indicated C-sections, either in cases of acute dystocia or scheduled in cases anticipating complications during parturition due to various reasons. Grouping was performed according to the criteria from previous studies (Frehner et al., 2018; Egloff et al., 2020; Rempel et al., 2021a; Rempel et al., 2021b; Frehner et al., 2021; Jungmann et al., 2022). PUI was defined as the complete failure of myometrial contractions to expel any neonates at term, although obstruction was ruled out by vaginal digital examination and/or X-ray. Bitches showed clear signs of stage one labor but no progression to stage two, with only weak or no uterine contractions. In contrast, bitches assigned to the OD group still exhibited strong uterine contractions, but after successful delivery of one or more puppies, labor was disturbed due to obstruction of the birth canal. The scheduled C-sections were further divided into bitches not in birth with serum P4 concentrations >2.5 ng/mL (NIB) and bitches already in birth (IB), showing serum P4 concentrations <2.5 ng/mL. All bitches underwent a thorough clinical and obstetrical examination. Blood samples for a complete blood cell count and determination of P4 were taken prior to surgery.

### 2.2 Study design, tissue sample collection, and processing

Two different sample sets were used. For both sample sets, interplacental uterine tissue was taken from periparturient bitches during medically indicated C-sections, as previously described (Frehner et al., 2018; Egloff et al., 2020; Rempel et al., 2021a; Rempel et al., 2021b; Frehner et al., 2021; Jungmann et al., 2023). After delivery of all puppies, a full-thickness interplacental biopsy, approximately 1.5 cm wide and 2 cm long, was taken from the incision line. If a sectio porro (C-section with subsequent ovariohysterectomy) was performed, the respective sample was taken after the removal of the uterus. The tissue was preserved according to its intended use.

Samples for organ bath and PGF2α ELISA were collected intentionally to be used in this study. One part of each sample was snap-frozen in liquid nitrogen and stored at −80°C until PGF2α determination by ELISA. Tissue intended for use in the organ bath was immediately transferred into a physiological (0.9%) saline solution and stored at 4°C until the start of the organ bath experiment. For organizational reasons, this storage time varied between 4 and 26 h. For the PGF2α ELISA, some well-characterized samples from a previous study were used additionally (Jungmann et al., 2023). Further information on the bitches, including breed, age, body weight, and serum P4 concentrations, is presented in Supplementary Table S1 in Supplementary Material.

For analyzing mRNA expression by real-time PCR (RT-RT-qPCR) and immunohistochemistry (IHC), a sample set of formalin-fixed paraffin-embedded (FFPE) well-classified interplacental and uteroplacental uterine tissue specimens, which has been used successfully in our previous studies (Frehner et al., 2018; Egloff et al., 2020; Rempel et al., 2021a; Rempel et al., 2021b; Frehner et al., 2021; Jungmann et al., 2022), was available. This sample set consisted of tissue samples from 12 bitches diagnosed with PUI and eight bitches with OD, which still showed strong uterine contractions. Grouping was based on strict criteria, as previously described (Frehner et al., 2018; Egloff et al., 2020; Rempel et al., 2021a; Rempel et al., 2021b; Frehner et al., 2021; Jungmann et al., 2022).

### 2.3 Organ bath experiment

Interplacental uterine tissue from n = 7 dogs (Supplementary Table S1) was subjected to motility studies in an organ bath. The preparation of tissue for the organ bath was performed as previously described (Jungmann et al., 2023). Briefly, following the removal of the endometrium, the circular and longitudinal myometrial layers were carefully separated using a stereomicroscope (Olympus SZ30 Stereomicroscope, Olympus Deutschland SE & Co. KG, Hamburg, Germany). Four strips, 1 cm long and 0.25 cm wide, aligned with the direction of the respective muscle fibers, were cut from each layer. The eight strips were transferred to the tissue chambers of the organ bath, filled with 12 mL of modified Krebs solution (in mmol/L: 1.2 MgCl_2_, 2.5 CaCl_2_, 1.2 NaH_2_PO_4_, 117 NaCl, 20 NaHCO_3_, 11 glucose, and 4.7 KCl). The Krebs solution was preheated at 37°C and constantly oxygenated with 95% O_2_ and 5% CO_2_ during the experiment. To record their contractility, strips were attached to an isometric force transducer (Hottinger Brüel & Kjaer GmbH, Darmstadt, Germany). The variations in tension were electrically transformed (Spider8 PC-Messelektronik, Hottinger Brüel & Kjaer GmbH) and recorded with computer software Catman^®^ Easy, Version 1.01 (Hottinger Brüel & Kjaer GmbH). A resting tension of 2 g (∼20 mN) was manually set for all strips, and the tissue was allowed to equilibrate for 60 min. The experiment was divided into timeframes (TFs) of 20 min. After a control period (TF1), stimulation with PGF2α was performed twice (TF 2 and 4) to check for the reproducibility of the response. For stimulation, the natural PGF2α dinoprost (Dinolytic ^®^ 5 mg/mL injectable solution, Zoetis Deutschland GmbH, Berlin, Germany) was used. One strip per layer was exposed to 50 μM, 0.5 µM, or 50 pM PGF2α or Krebs solution as a control. Each stimulation was followed by a washout period with Krebs solution (TF 3 & 5), and at the end of the experiment, 1 nM oxytocin (Oxytocin 10, I.E./mL, Injektionslösung, Serumwerk Bernburg AG, Bernburg, Germany) was used to confirm the vitality of the tissue strips (TF 6).

The experiments were analyzed as previously described for stimulation with oxytocin and denaverine hydrochloride (Jungmann et al., 2023). In every TF, the frequency (F) of contractions was counted, and for detailed analysis of the other parameters, up to ten contractions per TF were used. For each contraction, the peak value was determined, and together with the baseline tension before and after the contraction, the amplitude (A) and mean force (MF) of each contraction were determined. Additionally, the area under the curve (AUC), the integral of the time interval of one contraction, was used to measure the contractile effects of each PGF2α concentration. Results were transformed from g into mN (A and MF) or g·s into mN·s (AUC) by multiplying by 9.8. The individual values for A, MF, and AUC from the analyzed contractions per TF were averaged for the following statistical analysis. The response to the final stimulation with oxytocin was not analyzed in detail and only used to verify the vitality of the tissue.

### 2.4 Analysis of tissue PGF2α and serum P4 concentrations

PGF2α concentrations were determined by ELISA using snap-frozen interplacental full-thickness uterine tissues from six dogs included in the organ bath experiments (2.3.), as well as from seven dogs included in a previous study (Jungmann et al., 2023) (Supplementary Table S1). Dogs were assigned to one of three groups depending on the reason for the C-section following the criteria mentioned above: planned C-section NIB (n = 5), planned C-section IB (n = 5), and PUI (n = 3). Extraction of PGF2α from the uterine tissue was performed as previously described (Kowalewski et al., 2015b, Siemieniuch et al., 2014). After thawing, 30–50 mg of interplacental uterine tissue from each sample was mixed with 400 µL of 0.02% sodium acid in TBS, acidified by adding 45 µL of 1 N HCl. The tissue was homogenized using a TissueLyser II (QIAGEN GmbH, Hilden, Germany) for 5 min at 30 Hz. The homogenate was transferred to glass vials, and 3 mL of ethyl ether was added. The vials were vortexed thoroughly and incubated at −20°C for 4 h. Afterward, the supernatant was collected in another glass vial and evaporated to dryness under a stream of nitrogen while holding the vial in a 40 °C water bath. Finally, the sample was re-suspended with 400 μL TBS, mixed, and allowed to settle for 15 min at room temperature before being stored at −20°C until the ELISA was carried out.

For PGF2α determination, a commercial PGF2α high-sensitivity ELISA kit (ADI-931–069, ENZO Life Sciences, Inc., Farmingdale, NY, USA), already proven suitable for canine tissue (Kowalewski et al., 2015b), was used. The ELISA was carried out in accordance with the manufacturer’s instructions. Samples were diluted 1:100 and run in triplicate. The sensitivity of the assay given by the manufacturer was 0.98 pg/mL. The intra-assay coefficient of variability was 10.53%.

P4 was analyzed from a serum blood sample obtained before C-section using an enzyme-linked fluorescence assay (Mini VIDAS^®^, bioMérieux Germany, Nürtingen, Germany) (Brugger et al., 2011; Hussein et al., 2022).

### 2.5 Reverse transcriptase polymerase chain reaction and quantitative real-time PCR

For the identification of PTGS2 and PTGFR mRNA expression in the two myometrial layers, the previously described, well-characterized (Frehner et al., 2018; Egloff et al., 2020; Rempel et al., 2021a; Rempel et al., 2021b; Frehner et al., 2021; Jungmann et al., 2022) uterine FFPE tissue samples were used. FFPE blocks were cut into 8 µm sections, and layers were microscopically separated using a stereomicroscope (Olympus). The RNeasy ®FFPE Kit (Cat.No.73504, QIAGEN GmbH, Hilden, Germany) was used in accordance with the manufacturer’s instructions to isolate RNA from the paraffin-embedded tissue. The DNase digestion step using DNase I was included in the FFPE protocol. The resulting RNA concentration and quality were assessed using a spectrophotometer (NanoPhotometer^®^ NP80, Implen GmbH, München, Germany). RNA concentrations of 100 ng/μL were aimed at being used for cDNA synthesis. To adjust samples with too low concentrations to 100 ng/μL, the RNA was lyophilized in Lyovapor L-200 (BÜCHI Labortechnik GmbH, Essen, Germany) and re-suspended in the respective volume of RNase-free water. Full-length first-strand cDNA synthesis was carried out using M-MLV reverse transcriptase (RNase H minus, point mutant, Promega Corporation, Madison, WI, USA), as previously described (Körber et al., 2019; Rempel et al., 2021a). For quantitative real-time polymerase chain reaction (RT-qPCR), specific primer sets for PTGS2 and PTGFR and the reference genes ACTB and PTK2 were purchased from Microsynth (Microsynth AG; Balgach, Switzerland) (Table 1). The specificity of the primers was confirmed using BLAST http://blast.ncbi.nlm.nih.gov. (Rempel et al., 2021a; Jungmann et al., 2022; Körber and Goericke-Pesch, 2019). For RT-qPCR, 4 μL of cDNA (dilution 1:10) was combined with an 8 μL master mix (FastStart Essential DNA Green Master, Roche Diagnostics, Basel, Switzerland), 1 μL of the forward and reverse primers (10 pmol), and 1 μL of nuclease-free water. A LightCycler^®^ 96 real-time PCR system (software version 1.1.0.1320, Roche Diagnostics GmbH, Mannheim, Germany) with previously established cycling conditions was used (Körber and Goericke-Pesch, 2019; Rempel et al., 2021a), with all samples run in triplicate and a non-template control included in each assay. The PCR efficiencies of target and reference genes were calculated using a relative standard curve derived from a two-fold dilution series (1:2—1:128) of pooled cDNA samples run in triplicate. Evaluation of the RT-qPCR results was performed with a modified model based on the efficiency-corrected relative quantification according to Pfaffl (2001), also taking into account the expression of multiple reference genes (Hellemans et al., 2007), in this case, ACTB and PTK2.

**TABLE 1:** Sequence of primers for RT-qPCR and RT-PCR, amplicon length, efficiency, and accession number used for the investigation of mRNA expression in canine myometrium.

Primer	Accession No.	Forward sequence (5′→3′)	Reverse sequence (5′→3′)	Amplicon length (bp)	Efficiency
*PTGS2*	NM_001003142	GGA​GCA​TAA​CAG​AGT​GTG​TGA​TGT​G	AAG​TAT​TAG​CCT​GCT​CGT​CTG​GAA​T	88	2.01
*PTGFR*	NM_001048097.1	CAGTGCCCT GGTAATCACAG	GCG​GAT​CCA​GTC​TTT​ATC​GG	91	^a^
*ACTB*	NM_001195845.3	GCT​GTG​CTG​TCC​CTG​TAT​G	GCG​TAC​CCC​TCA​TAG​ATG​G	98	2.1
*PTK2*	XM_038685127.1	AGA​TGC​TGA​CCG​CTG​CTC​AT	TCA​GTG​TGG​CCT​CGT​TGG​TC	104	1.86

^a^ Creation of standard curve for RT-qPCR was not successful; consequently, primers were used for RT-PCR only.

Expressions of PTGFR and the reference gene PTK2 in fresh canine uterine uteroplacental tissue, including endometrium and parts of the placenta, were proven by standard RT-PCR. A freshly frozen sample of uteroplacental tissue was used for mRNA extraction (SV Total RNA Isolation System, Promega), and cDNA was written as described above. Additionally, FFPE-derived cDNA from both myometrial layers, already prepared for RT-qPCR, was used. To a master mix containing 2 μL of MgCl_2_, 4 µL of PCR buffer, 32.75 µL of nuclease-free water, 0.5 μL of forward primer (10 pmol/μL), 0.5 μL of reverse primer (10 pmol/μL), 0.25 μL of GoTaq^®^ Flexi DNA Polymerase (Promega), and 10 μL of cDNA or water as a non-template control were added. Cycling conditions were initially 95°C for 10 min, followed by 35 cycles of 1 min at 94°C, 2 min at 60°C, 1 min 30 s at 72°C, and, finally, 72°C for 10 min. The amplicon size was evaluated by gel electrophoresis.

### 2.6 Immunohistochemistry for PTGFR and evaluation of staining

For IHC, our established protocol was used (Rempel et al., 2021a; Rempel et al., 2021b; Jungmann et al., 2022). The paraffin-embedded uterine tissue was cut in serial sections of 3 µm thickness and mounted on SuperFrost-Plus slides (Menzel Glaser, Braunschweig, Germany). Slides were de-paraffinized in xylol and rehydrated in decreasing ethanol concentrations, followed by heat-induced antigen retrieval in 0.01 M sodium citrate buffer at pH 6 and blocking of endogenous peroxidase with 3% hydrogen peroxide. Non-specific protein binding was blocked using 10% goat serum with 5% bovine serum albumin in ICC buffer Na_2_HPO_4_, KH_2_PO_4_, KCl, and NaCl with 0.3% Triton X, pH between 7.2 and 7.4. Slides were then incubated overnight with the PTGFR rabbit polyclonal primary antibody (ab203342, dilution 1:80, corresponding to 0.013 μg/μL; Abcam plc, Cambridge, United Kingdom). Additionally, an isotype control with an irrelevant rabbit IgG (I-1000 Control Antibody, Vector Laboratories, Burlingame, CA, USA) in the respective protein concentration and a negative control using ICC buffer only were prepared. Human placental tissue was used as a positive control. On the following day, after washing three times with the ICC buffer, samples were incubated with the secondary antibody (BA-I-1000–5, Vector Laboratories) in 10% goat serum. The immunopositive signal was visualized with an immunoperoxidase method in accordance with the manufacturer’s instructions (VECTASTAIN PK-6100 ABC-Elite Standard: HRP and Vector Nova-RED Substrate Kit SK-4800, Vector Laboratories).

Staining was analyzed by a blinded evaluator using a light microscope (Olympus BX 45, Olympus Europa SE & Co. KG, Hamburg, Germany). The localization of the immunopositive signals was evaluated descriptively. In addition, the staining intensity of the longitudinal and circular myometrial layers was scored separately and graded semi-quantitatively according to an ordinal score system (1: weak, 2: moderate, and 3: strong staining). The blinded evaluator scored all slides twice, and Cohen’s kappa value (Jakobsson et al., 2005) was used to assess the inter-examination agreement.

### 2.7 Statistical analysis

All data were organized using Microsoft 365 MSO (Version 2,309, Microsoft Corporation, Redmond, WA, USA). For statistical tests and data presentation, GraphPad Prism9 software (version 9.0.0, GraphPad Software, Inc., La Jolla, CA, USA) was used, if not stated otherwise. If necessary, all data were initially tested for normal distribution using the Shapiro–Wilk test. All data were presented as the mean ± standard deviation, if not stated otherwise [x ± SD]. Statistical differences were considered significant at a level of *p* ≤ 0.05.

#### 2.7.1 Organ bath

To assess the effect of PGF2α stimulation on the parameters of contraction, namely, A, MF, AUC, and F, in an organ bath, a two-way repeated measurement (RM) ANOVA was used. For each myometrial layer separately (factor “layer”), the response to the three different tested concentrations of PGF2α (factor “concentration”) in the two stimulations (factor “stimulation cycle”) was analyzed. Furthermore, the responses to the different concentrations were compared between layers. RM-ANOVA was analyzed for simple main effects of the respective two independent factors and interactions between them. For *post hoc* comparison, Tukey’s multiple comparison test was used, investigating the effect of PGF2α stimulation compared to untreated controls and comparing responses between the three different concentrations. Additionally, Šídák’s multiple comparison test was used to compare the responses during the two stimulation cycles and between layers. To rule out a possible influence of the covariant factors, storage time of samples, serum P4 concentration, and age of bitches on the response to stimulation, multivariate analysis of covariance with SAS (SAS Studio 3.81, SAS Institute, Cary, NC, USA) was utilized.

#### 2.7.2 Tissue PGF2α concentrations and correlation with serum P4

PGF2α concentrations in the uterine tissue homogenates were analyzed by comparing the respective groups (PUI; IB and NIB). As all data were normally distributed, an ANOVA was performed, followed by Tukey’s multiple comparison test if the ANOVA revealed significant differences (*p* < 0.05). Additionally, we aimed to perform a correlation analysis between uterine PGF2α concentrations and peripheral serum P4 concentrations. As blood samples for P4 analysis were only available from two (of three) bitches in the PUI group, statistical analysis was only performed to compare NIB and IB samples. An unpaired *t*-test was used to compare the normally distributed data on P4 concentrations. Similarly, correlation analyses between PGF2α and P4 concentrations were only performed in IB and NIB samples. Due to the non-normally distributed data, a two-tailed Spearman test was performed.

#### 2.7.3 RT-qPCR for PTGS2

For the analysis of PTGS2 gene expression, log transformation of ratio data was performed, revealing a normal distribution of log-transformed data according to the Shapiro–Wilk test. Subsequently, t-tests were used for comparison of the circular and longitudinal myometrial layers within and between PUI and OD using the respective (raw/log-transformed) datasets.

#### 2.7.4 PTGFR immunohistochemistry

A statistical analysis of PTGFR staining was performed on interplacental samples. Due to the small number of uteroplacental samples, PTGFR staining of uteroplacental samples was analyzed only descriptively. The high inter-exam agreement for staining results (κ = circular layer: 0.93; longitudinal layer: 0.84) allowed the use of mean values of both examinations for further statistical comparison of myometrial layers (circular/longitudinal) within and between groups (PUI/OD). Furthermore, the staining intensity of all circular samples was compared to all longitudinal staining results, regardless of dystocia groups. As none of the datasets were normally distributed following the Shapiro–Wilk test, either a Wilcoxon matched-pairs signed rank test for paired data or a Mann–Whitney test for unpaired data was carried out. Data were presented as geometric mean and dispersion factor [xg (DF)].

## 3 Results

### 3.1 Organ bath experiment

**FIGURE 1:**
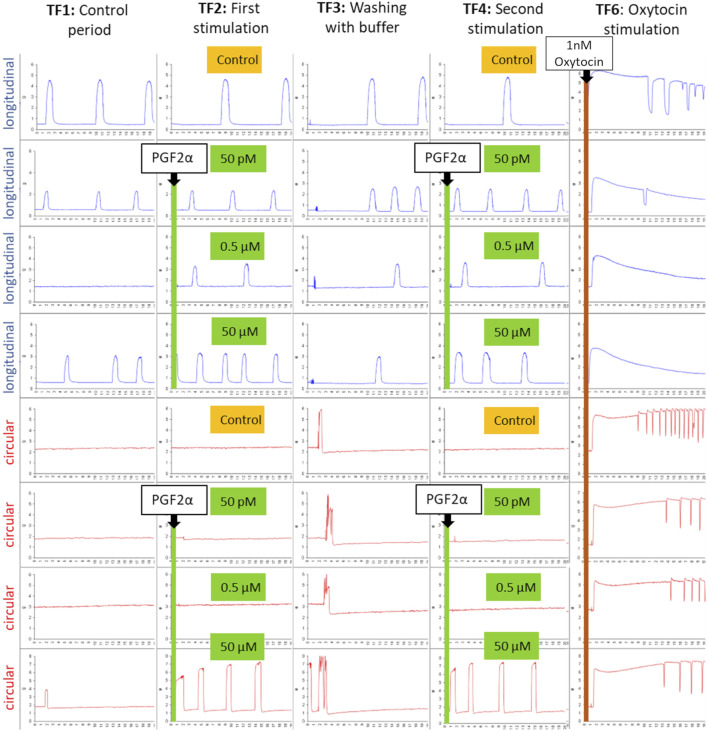
An example of the typical contraction patterns of isolated canine myometrium following stimulation with PGF2α. Four strips of the longitudinal (blue) and circular (red) layers were mounted in the organ bath, recording spontaneous contractions (TF1). (TF2) Strips were stimulated with different concentrations of PGF2α (50 pM, 0.5 µM, 50 µM) and afterward (TF3) washed with the buffer. PGF2α stimulation in the respective concentration was repeated (TF4), as was the washout (TF5). Finally, strips were stimulated with 1 nM oxytocin to prove their vitality (TF6). A reproducible increase in contractility in the circular layer can be observed following stimulation with 50 µM PGF2α (line 6, TF2/TF4).

In the seven organ bath experiments, a total of 56 tissue strips were used. Figure 1 demonstrates an example of the responses of the separated myometrial layers to stimulation with PGF2α from one experiment.

Although not every strip exhibited spontaneous contractions in the control period (TF1), the final application of 1 nM oxytocin in TF 6 confirmed the vitality of all strips. Subjectively, the circular layer responded with a visible increase in contractility only to 50 µM PGF2α, whereas the other concentrations did not have any effect at all. The response ceased with the washout period and was reproducible when stimulated for a second time. In contrast, the longitudinal layer showed no visible effect on any of the tested PGF2α concentrations.

**FIGURE 2:**
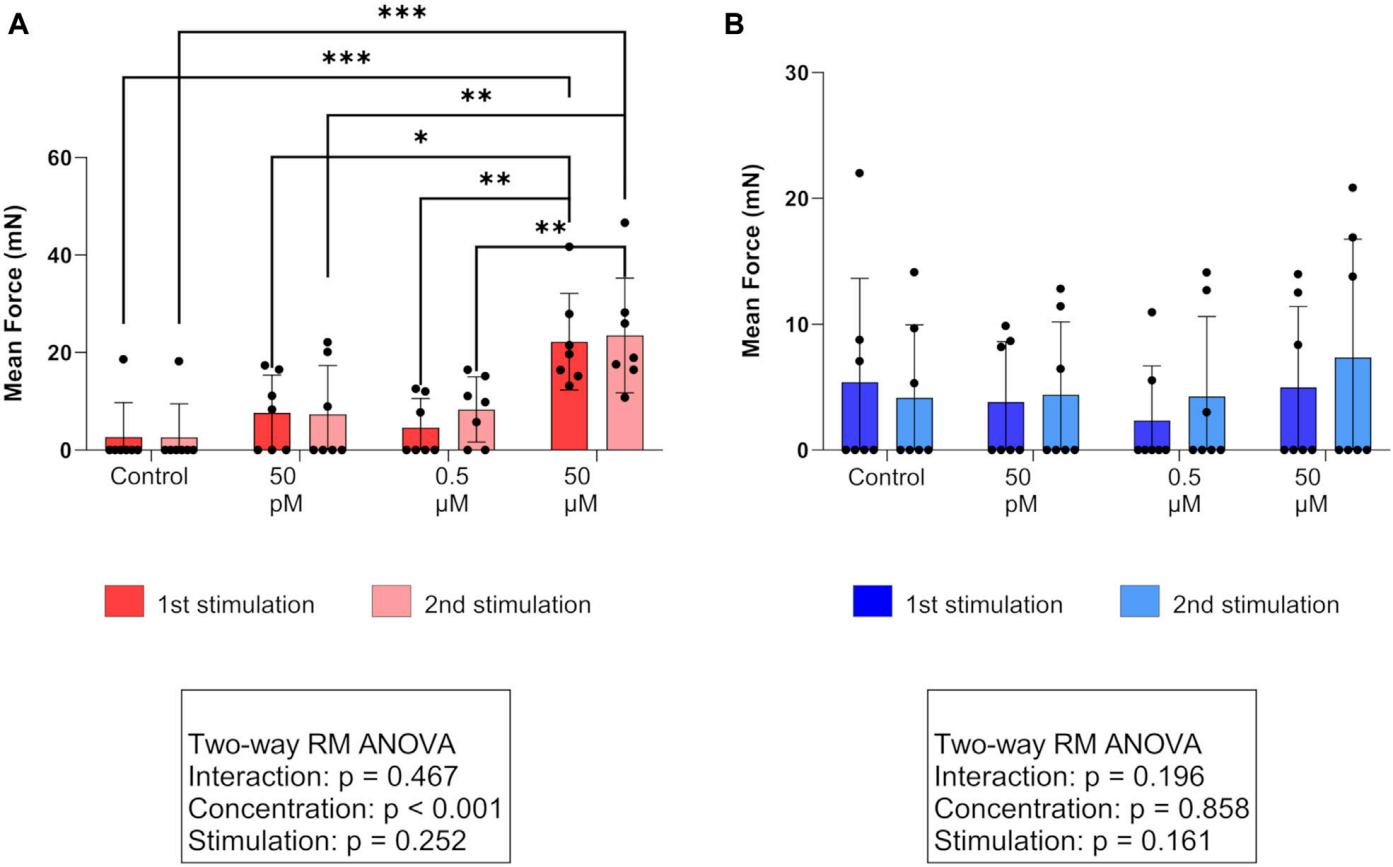
Effect of PGF2α stimulation on the mean force of contractions in **(A)** the circular (red) and **(B)** longitudinal (blue) myometrial layers of the canine parturient uterus *in vitro*. Strips were stimulated with 50 pM, 0.5 µM, and 50 µM PGF2α or remained untreated as a control. The results are presented as the mean ± standard deviation. Bars with asterisks differ significantly (*p ≤ 0.05; **p ≤ 0.01; ***p ≤ 0.001).

For A, MF, and AUC, a two-way RM ANOVA revealed no interaction between the “stimulation cycle” and “concentration” in either of the layers. However, in the circular layer, simple main effects analysis confirmed a significant effect (*p* < 0.01, each) of the used PGF2α concentration on the three observed parameters. Accordingly, stimulations with 50 µM PGF2α significantly increased A, MF (Figure 2A), and AUC of contractions in both stimulation cycles in the circular layer compared to untreated controls (*p* < 0.01, each). Investigating the different PGF2α concentrations used, the effect of 50 µM PGF2α was superior to 0.5 µM and 50 pM (*p* < 0.05, each) with one exception. Specifically, when comparing the effects of 50 μM and 50 pM PGF2α stimulation on A, there was only a trend toward significance in the first stimulation (*p* = 0.087). The observed effects of PGF2α stimulation in the circular layer did not differ significantly between stimulation cycles, indicating the reproducibility of the responses. The F of contractions was not significantly affected by PGF2α administration, although an increase in contractile frequency was visible following stimulation with 50 µM PGF2α.

In the longitudinal layer, we identified no statistically significant effect of any PGF2α concentration (Figure 2B).

Direct comparison of layers revealed a significantly higher A and MF in the circular layer following stimulation with 50 µM PGF2α in both stimulation cycles (A: *p* < 0.05, both; MF: *p* < 0.01, both). This explains the results of the simple main effects analysis, where a significant effect of the factor “layer” (A: *p* < 0.05; MF: *p* < 0.01) was found when comparing the responses to 50 µM between layers. For all PGF2α concentrations used, the resulting AUC did not differ significantly between layers.

Multivariate analysis of the covariance of the factors storage time, serum P4, and age of bitches at C-section showed no effect of these three factors on A, MF, and AUC. Surprisingly, the F of contractions was significantly affected by the storage time (*p* < 0.001). However, this effect was not visible in the spontaneous activity of the strips. After stimulation with 50 µM PGF2α, the F of contractions generally increased independent of the storage time, but this increase was significantly more pronounced with shorter storage time.

### 3.2 Peripheral P4 and local PGF2α concentrations

**FIGURE 3:**
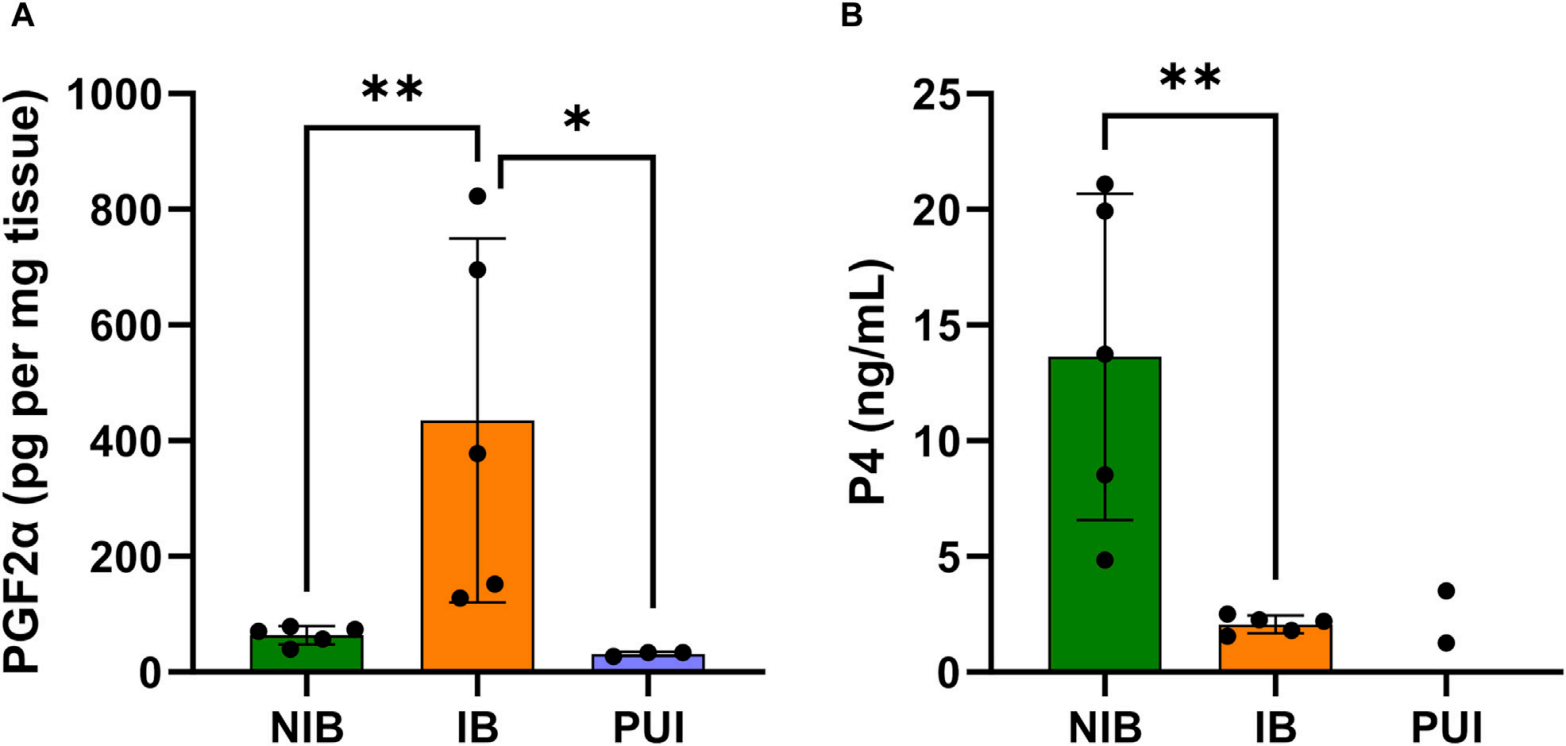
PGF2α concentrations in interplacental uterine tissue determined by ELISA analysis **(A)** and serum progesterone (P4) concentrations **(B)** comparing tissue samples from bitches not in birth (NIB (green), P4 > 2.5 ng/mL) and in birth (IB (orange), P4 < 2.5 ng/mL). PGF2α concentrations were further assessed in bitches presented with primary uterine inertia (PUI (blue)). Results are presented as the mean ± SD. Bars with asterisks differ significantly (*p ≤ 0.05; **p ≤ 0.01).

The determination of PGF2α concentrations in full-thickness interplacental uterine tissue samples revealed significantly higher PGF2α concentrations in samples from bitches in labor with P4 < 2.5 ng/mL (IB group) compared to bitches with P4 > 2.5 ng/mL not in birth (NIB Group, *p* < 0.036, Figure 3A). A comparison of PGF2α concentrations from samples obtained from IB and PUI bitches revealed significantly lower PGF2α concentrations in bitches diagnosed with PUI (*p* = 0.047, Figure 3A). However, PGF2α concentrations did not differ significantly between PUI and NIB.

By definition, serum P4 concentrations from bitches that had not started labor (NIB) were significantly higher compared to the IB group (*p* = 0.006, Figure 3B). The Spearman test identified a negative correlation between local PGF2α concentrations in the uterus and serum P4 concentrations (r = −0.661; *p* = 0.044).

### 3.3 mRNA expression of *PTGS2* and *PTGFR*

In both myometrial layers, *PTGS2* mRNA expressions (ratio) as assessed by RT-qPCR did not differ significantly between PUI and OD. Likewise, no significant differences were found when comparing *PTGS2* ratios from all longitudinal samples with all circular tissue samples.

For PTGFR, it was not possible to generate an adequate standard curve, thus, no consecutive RT-qPCR analysis of PTGFR mRNA expression was possible. RT-PCR confirmed PTGFR expression in fresh frozen canine uteroplacental tissue (Supplementary Figure S1, in Supplementary Material). Nonetheless, using the FFPE-derived cDNA, no distinct amplicons could be identified in gel electrophoresis.

### 3.4 Staining for PTGFR

All samples showed specific immunostaining for PTGFR. In the endometrium, the epithelial cells and uterine glands stained positively for PTGFR.

**FIGURE 4:**
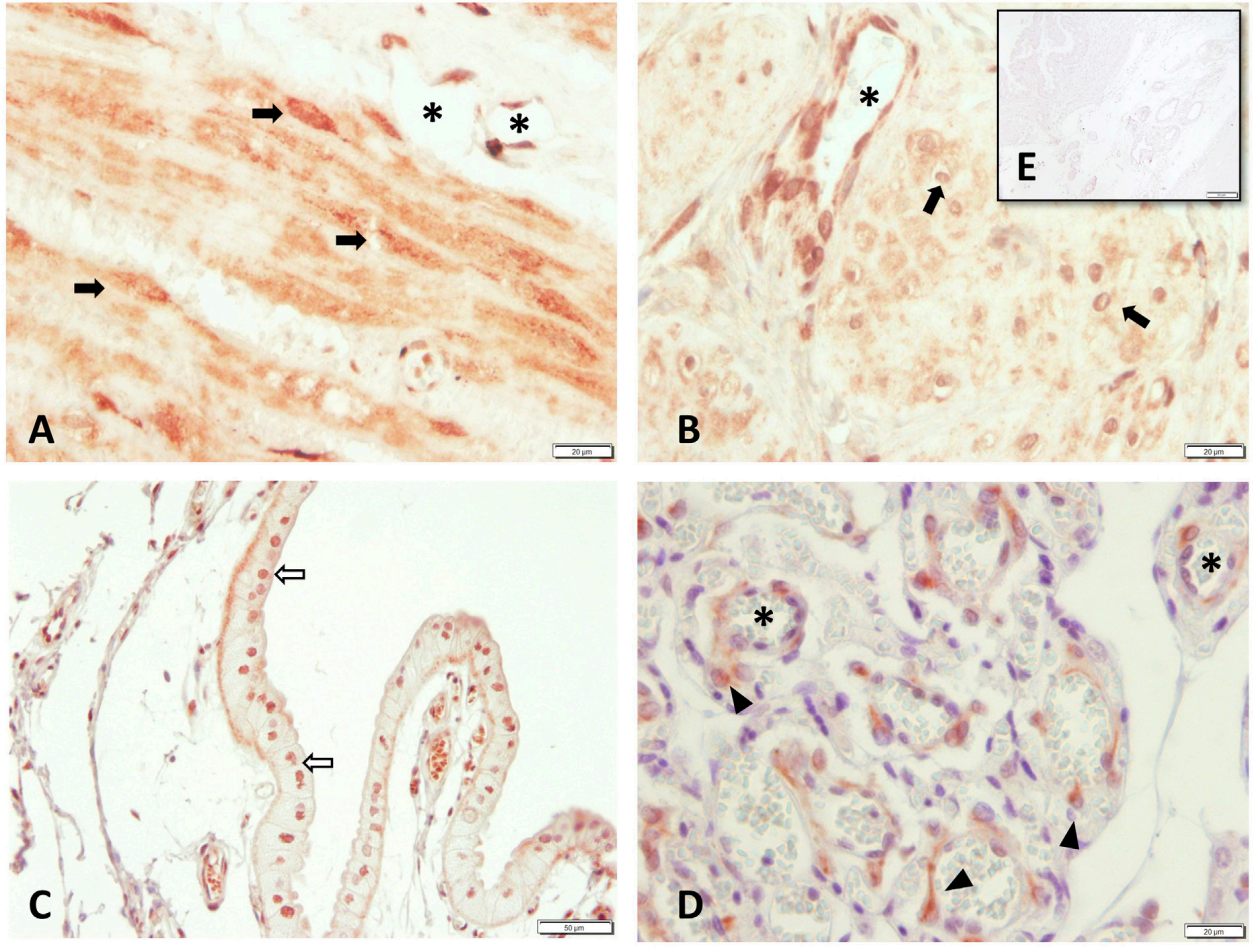
PTGFR protein localization as revealed by immunohistochemical staining in canine uterine tissue **(A–C**+**E)** interplacenta, IP; **(D)** uteroplacenta, UP. **(A)** and **(B)** myometrium: **(A)** circular layer, **(B)** longitudinal layer), **(C)** uterine luminal epithelium, **(D)** placental labyrinth, and **(E)** isotype control for PTGFR given as insert. Symbols indicate specific cell types: (➨) myocytes, (➪) uterine luminal epithelial cells, (*) blood vessels, (▲) maternal decidual cells.

Intense staining for PTGFR was identified in the endothelial cells of blood vessels and myocytes in both the longitudinal and circular layers of the myometrium (Figure 4). In the placenta, staining was observed in fetal trophoblast cells; however, staining intensity and distribution varied between animals. Maternal decidual cells showed only weak and/or sporadic PTGFR signals.

**FIGURE 5:**
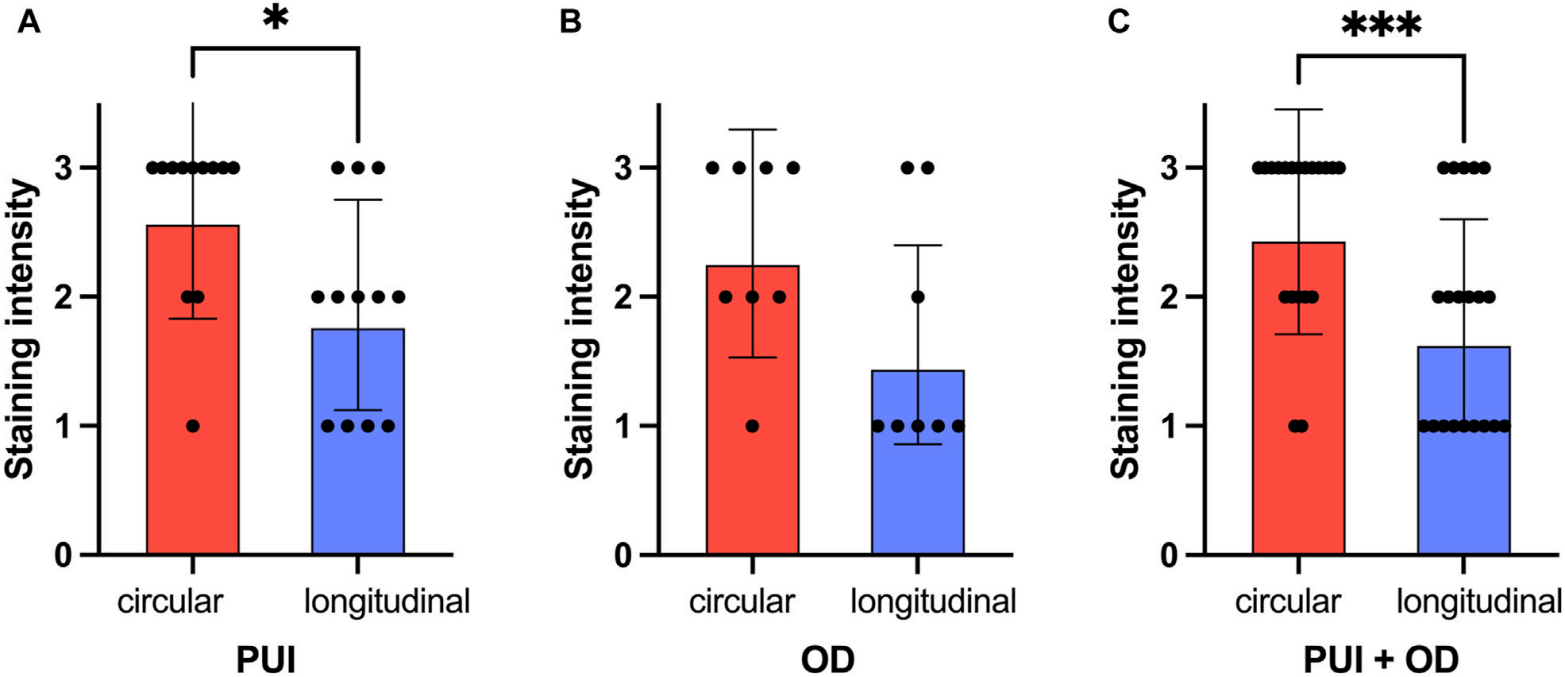
Comparison of PTGFR immunohistochemical staining intensity (0 = no staining; 3 = strong staining) between myometrial layers (circular/longitudinal) for **(A)** PUI (n = 12) and **(B)** OD (n = 8), respectively, and **(C)** regardless of the group (PUI + OD). Results are presented as the geometric means with the dispersion factor. Asterisks indicate significant differences (*p < 0.05; ***p ≤ 0.001).

When comparing the staining intensity between both myometrial layers in interplacental tissue independent of groups, significantly stronger PTGFR staining was observed in the circular layer than in the longitudinal layer (*p* < 0.001, Figure 5C). Similar observations were made when looking at the individual groups, with the difference between layers being only significant for samples obtained from PUI bitches (*p* = 0.016) (Figures 5A, B).

## 4 Discussion

Currently, the effect of prostaglandins, especially PGF2α, in parturition and particularly in the initiation and maintenance of uterine contractions in dogs is not fully understood. In non-pregnant bitches with open-cervix pyometra, PGF2α (natural/synthetic) is described as a conservative treatment option to promote the expulsion of uterine contents (Nelson et al., 1982; Meyers-Wallen et al., 1986; Günzel-Apel and Bostedt, 2016), assuming ecbolic/uterotonic activities. Moreover, PGF2α, or a combination of PGF2α and aglepristone, can be used to terminate pregnancies (Wichtel et al., 1990; Hubler et al., 1991; Romagnoli et al., 1991; Hoffmann et al., 1999). PGF2α, due to its action on smooth muscle cells, is known to cause dose-dependent severe side effects such as tachycardia, tachypnea, salivation, vomiting, diarrhea, and occasionally seizures or even collapse and death (Plumb, 2002; Kustritz and Hess, 2007). The aim of our current study was to gain deeper insights into the role of PGF2α on the canine parturient myometrium and assess its ecbolic capacities to activate uterine contractions *in vitro*. Experiments in the organ bath identified an effect of high dosages of PGF2α (50 µM); however, this effect was observed only on the circular myometrial layer. Our findings from the organ bath experiments contrast with those of Gogny et al. (2010), who described a concentration-dependent (10 nM–10 µM) contractile effect of PGF2α in circular and longitudinal myometrial fibers. Moreover, Gogny et al. identified a significantly higher responsiveness to PGF2α of longitudinal fibers from bitches in anestrus or metestrus receiving aglepristone treatment three to five days before ovariohysterectomy. However, the bitches used by Gogny et al. were non-pregnant and in metestrus or anestrus, and they lacked endogenous PGF2α production by placental trophoblast cells as well as other endocrine and physiological changes that distinguish the late-term and parturient uterus from other reproductive stages. Different responses to PGF2α depending on the cycle stage and/or pregnancy status have been described earlier for other species; for example, and similar to our results, a higher responsiveness to PGF2α was described in the circular myometrial layer compared to the longitudinal layer in rats at term (Tuross et al., 1987). Differences in response between the two myometrial layers are not surprising and were previously shown in the same experimental setup following oxytocin stimulation (Jungmann et al., 2023). During physiological parturition, interaction between both myometrial layers is essential, whereby both layers have slightly different functions and thus seem to have a differential responsiveness to stimulating agents. PGF2α interacts with its specific receptor, PTGFR. As a member of the G protein-coupled receptor family, PTGFR can be desensitized and downregulated by its own ligand (Watanabe et al., 1994; Ferguson et al., 1998; Xu et al., 2013). The reduction in PTGFR availability by increasing PGF2α concentrations around parturition could explain the low responsiveness of the myometrium to PGF2α stimulation observed in the organ bath. In IHC, we found a significantly higher PTGFR expression in the circular layer of the samples compared to the longitudinal layer, independent of the underlying reason for C-section (PUI or OD). This indicates that a higher PTGFR expression in the circular myometrial layer is a general parturition-associated phenomenon. In addition, this might explain, at least to some extent, why in the organ bath the response to PGF2α stimulation was restricted to the circular myometrial layer. Relative overexpression of PTGFR in the circular layer allows for the observed contractile response to at least high concentrations of PGF2α. Nevertheless, a larger number of bitches and a more homogeneous population sampled in the periparturient period, namely, prepartal, parturient, and post-parturient, would have been beneficial and would have allowed for even deeper insights into the role of PGF2α on the canine myometrium during parturition. However, neither age nor P4 concentration affected the investigated organ bath parameters, thus supporting our results in general. Different from this, the variable storage time of samples before processing affected the frequency of contractions. That is, the increase in the frequency of contractions after 50 µM PGF2α application in the circular layer was more pronounced after short-term storage. This seems plausible, as tissue quality cannot remain unaffected by increasing storage time. However, storage was necessary from an organizational point of view (with many C-sections being performed at night and laboratory availability restricted to the daytime). In contrast, in our previous study, no effect of storage time on the response to oxytocin stimulation was determined (Jungmann et al., 2023), and neither were the parameters A, MF, and AUC influenced by storage time in the present study.

An increased relative *PTGFR* mRNA expression was reported using uteroplacental tissue samples, not only during preimplantation but also during prepartum luteolysis compared to mid-gestation (Kowalewski et al., 2010). During prepartum luteolysis, positive signals for *PTGFR* mRNA expression were localized by situ hybridization in all trophoblast cells and endometrial glandular chambers (Kowalewski et al., 2010), so increased expression of *PTGFR* in these compartments is likely. Nevertheless, the situation in the parturient canine myometrium remains unclear. Up until date, the myometrial distribution of *PTGFR* has never been investigated in detail by distinguishing between myometrial layers. In IHC, we confirmed the protein expression of PTGFR in both myometrial layers with a stronger staining intensity in the circular myometrial layer; thus, quantitative analysis of receptor expression at the mRNA level should be performed. Rempel et al. (2021a) investigated *PTGFR* expression in PUI and OD but failed to identify significant differences. These authors, however, used full-thickness uterine samples from interplacental and uteroplacental sites, with the latter including placental tissue. In our study, the myometrial layers were carefully separated from adherent connective tissue, endometrium, and placenta. Unfortunately, the determination of *PTGFR* expression in FFPE-derived mRNA was not successful. The use of FFPE tissue is known to be challenging, as the process of fixation and paraffin embedding often leads to RNA degradation and chemical alterations (Guo et al., 2016). Nonetheless, gene expression of reference genes and *PTGS2* was measurable in all samples, indicating that the mRNA integrity was sufficient for more abundantly expressed genes. An additional RT-PCR using cDNA from fresh, frozen uteroplacental uterine tissue and the analysis of *PTGFR* mRNA expression carried out by Rempel et al. (2021a) prove the existence of *PTGFR* in the tissue. As quantitative analysis of myometrial *PTGFR* expression in dogs in the two layers is of great interest in the context of myometrial contractile activity, further studies should use uterine tissue that is immediately dissected into layers and frozen as soon as possible.

*PTGS2* mRNA expression (ratio), the rate-limiting factor for prostaglandin synthesis, was also investigated in the canine pregnant uterus (Rempel et al., 2021a). *PTGS2* expression did not differ between PUI and OD independent of tissue localization (interplacental versus uteroplacental tissue), but no conclusions could be drawn regarding layer-specific mRNA expression due to the full-thickness biopsies used. With respect to the previous IHC results for PTGS2, where the longitudinal myometrial layer stained significantly stronger than the circular layer, independently of the reason for the C-section (Rempel et al., 2021a), we anticipated differing mRNA expressions between both individual myometrial layers. The observed lack of differences in *PTGS2* mRNA expression when comparing layers combined with the results of Rempel et al. (2021a) might indicate regulation on a post-transcriptional level and supports that, although crucial for prostaglandin supply and initiation of parturition in dogs (Kowalewski et al., 2010; Kowalewski et al., 2014), alterations in *PTGS2* expression are not likely to be involved in the pathophysiology of canine UI. Interestingly, in human myometrial cells, a PGF2α-mediated positive feedback mechanism for PTGFR-induced PTGS2 expression has been shown (Xu et al., 2013). Thus, PGF2α is able to overcome the rate-limiting effect of PTGS2 in prostaglandin synthesis.

Although the opposing behavior of circulating levels of serum P4 and PGF2α at term is well-described in periparturient bitches (Concannon et al., 1988; Nohr et al., 1993; Hoffmann et al., 1994; Luz et al., 2006; Kowalewski et al., 2010), little is known about the actual PGF2α concentrations in uterine tissue as the target organ during labor. Currently, based on *in vitro* and gene expression studies, the placenta, namely, the fetal trophoblast, is considered to be the major source of PGF2α (Luz et al., 2006; Kowalewski et al., 2010; Gram et al., 2013), despite evidence that the periparturient canine endometrium is also able to produce significant amounts of PGF2α *in vitro* (Luz et al., 2006). To the best of our knowledge, our study is the first to investigate PGF2α concentrations in uterine interplacental tissue obtained from bitches at term. As expected, we identified a negative correlation between local uterine PGF2α and peripheral P4 concentrations in serum. PGF2α concentrations were significantly higher in tissue obtained from bitches already in labor (IB, P4 < 2.5 ng/mL, indicating active luteolysis) than in bitches with still elevated P4 serum levels (NIB). Interplacental concentrations identified in the IB group (mean ± SD: 435.24 ± 281.51 pg/mg) were lower than levels obtained from endometrial explants at days 63 and 64 of pregnancy (mean ± SEM: 846 ± 118 pg/mL/mg), although P4 concentrations were not described in the latter study (Luz et al., 2006). Despite the lower concentrations, possibly related to the different tissue composition (full thickness versus endometrium only) and the method, our results clearly confirm the uterus, not only the placenta, as a relevant production site of PGF2α in the immediate prepartum period. Additionally, lower PGF2α concentrations were determined in PUI compared to IB, similar to NIB. Unfortunately, a correlation analysis was not possible, as the respective P4 levels were only available from two of the three PUI bitches and the generally low number of PUI animals. Whether this decrease in PGF2α levels is time-dependent, as bitches diagnosed with PUI are in labor longer than bitches scheduled for an elective C-section, or whether it is related to the occurrence of PUI requires further investigation with a larger number of animals. Additionally, the determination of uterine PGF2α concentrations from bitches within undisturbed labor that already gave birth to at least one puppy would be highly interesting to understand the course of PGF2α concentrations during parturition. Unfortunately, it is not possible to create a control group that would be of great interest in all the experiments conducted in this study because it is not feasible to take samples from clinically healthy periparturient dogs. Even if it is not completely transferable, a comparison of the average measured PGF2α concentration in the IB group with the PGF2α concentrations applied to the tissue in the organ bath revealed that the actual concentration corresponded to the dose of 50 pM. However, in the organ bath, 50 pM had no obvious effect on myometrial contractions. The dose able to achieve at least an effect in the circular layer is thus almost 98,000 times higher than the naturally occurring PGF2α concentrations measured in uterine interplacental tissue during prepartum luteolysis. Assuming that PGF2α promotes uterine contractions during labor, the corresponding dosage is supposed to show an effect *in vitro*. All results of this study question the direct uterotonic effect of PGF2 on canine myometrium at term. Nevertheless, future studies should investigate local PGF2α concentrations in interplacental and uteroplacental sites, as well as in the placenta, and perform *ex vivo* organ bath experiments with PGF2α stimulation in uteroplacental tissues as well as in interplacental tissues obtained from bitches with high P4. Studies on mice (Sugimoto et al., 1997) and humans (Xu et al., 2013) showed that PGF2α stimulates prepartal OXTR expression, presenting a trigger for active labor. PGF2α also stimulates the formation of gap junctions in the myometrium, improving cell–cell communication and, thus, enabling coordinated, strong myometrial contractions (Puri and Garfield, 1982; Xu et al., 2013; Xu et al., 2015). In addition, PGF2α is known to enhance the effects of oxytocin and corresponding agonists, presumably by providing Ca^2+^ from a pool inaccessible to oxytocin (Fuchs, 1995). Our data support these assumptions that the contractile effect of PGF2α might be predominantly indirect.

## 5 Conclusion

Our study could not prove an overall stimulatory effect of PGF2α on pregnant canine myometrium at term *in vitro*. High doses of this compound in the organ bath increased all contraction parameters except the F in the circular layer but not in the longitudinal layer, corresponding with higher PTGFR protein expression in the circular layer. However, the requirement for an effective uterotonic substance is likely to activate both layers, possibly ensuring functional labor. Downregulation of PTGFR following increased PGF2α release during prepartum luteolysis might be the reason for the observed low responsiveness of the myometrium to PGF2α treatment *in vitro*. Concentrations of PGF2α in the uterus were negatively correlated with serum P4 and were the highest during prepartum luteolysis, while contractile myometrial activity was shown to steadily increase until expulsion of the first puppy (van der Weyden et al., 1989). In conclusion, the tested PGF2α concentrations did not directly activate uterine contractions of both myometrial layers of the parturient bitch in an organ bath approach. Consequently, we suggest that PGF2α might affect uterine contractility mainly indirectly through the activation of different contraction-associated mechanisms. Further research is required to identify the underlying mechanisms.

## Data Availability

The original contributions presented in the study are included in the article/Supplementary Material; further inquiries can be directed to the corresponding author.
