# Supplementary material for: Insights into the role of PGF2α in canine periparturient myometrium
Source: Front Physiol. 2024 May 28;15:1392080. doi: 10.3389/fphys.2024.1392080 (PMC11165301; doi:10.3389/fphys.2024.1392080)
Supplement: Supplementary file 1 [file Table1.DOCX]

Supplementary Material

Supplementary Table S1: Overview of all dogs included in the study. Dogs are included in the PGF2α organ bath (O) experiment and/or the ELISA (E) for determination of PGF2α concentrations in the uterine tissue. Detailed information about the bitches including breed, serum progesterone concentration at the examination before surgery (P4; in ng/mL), age (in years), body weight (in kg), the total number of all puppies in the litter, and the reason for the C-section (PUI = primary uterine inertia; SUI = secondary uterine inertia) is given. Reasons for elective C-section were breed of the bitch, singleton pregnancy, or pregnancy complications.

| Breed | P4 (ng/mL) | Age (years) | Body weight (kg) | Total number of puppies | Reason for C-section |
| --- | --- | --- | --- | --- | --- |
| Golden Retriever | 1.8 | 4.0 | 33.3 | 5 | Elective C-section |
| Kooikerhondje | 1.7 | 4.2 | 8.8 | 6 | PUI |
| Husky | * | 4.3 | 23.3 | 3 | Uterine rupture |
| Tervueren | 3.5 | 7 | 18.0 | 5 | PUI |
| Leonberger | 1.6 | 2.3 | 71.3 | 12 | Elective C-section |
| Staffordshire Bullterrier | 1.5 | 3.5. | 14.9 | 1 | Elective C-section |
| Haverneser | 1.3 | 1.5 | 10.9 | 9 | PUI |
| Pomeranian | 21. | 5.2 | 2.6 | 1 | Elective C-section |
| Boston Terrier | 13.7 | 6.0 | 9.4 | 1 | Elective C-section |
| Boston Terrier | 8.5 | 2.8 | 9.1 | 5 | Elective C-section |
| Great Swiss Mountain Dog | 4.8 | 4.5 | 49.7 | 4 | Elective C-section |
| Briard | 19.9 | 3.9 | 33.6 | 1 | Elective C-section |
| Parson Jack Russel Terrier | 2.2 | 4.2 | 7.8 | 4 | Elective C-section |
| Miniature Bullterrier | 2.5 | 4.9 | 17.8 | 3 | Elective C-section |
| Miniature Bullterrier | 2.3 | No data | 14.2 | 5 | Elective C-section |
| Mongrel | * | 10.5 | 11.9 | 5 | PUI |

**Supplementary Figure S1.**


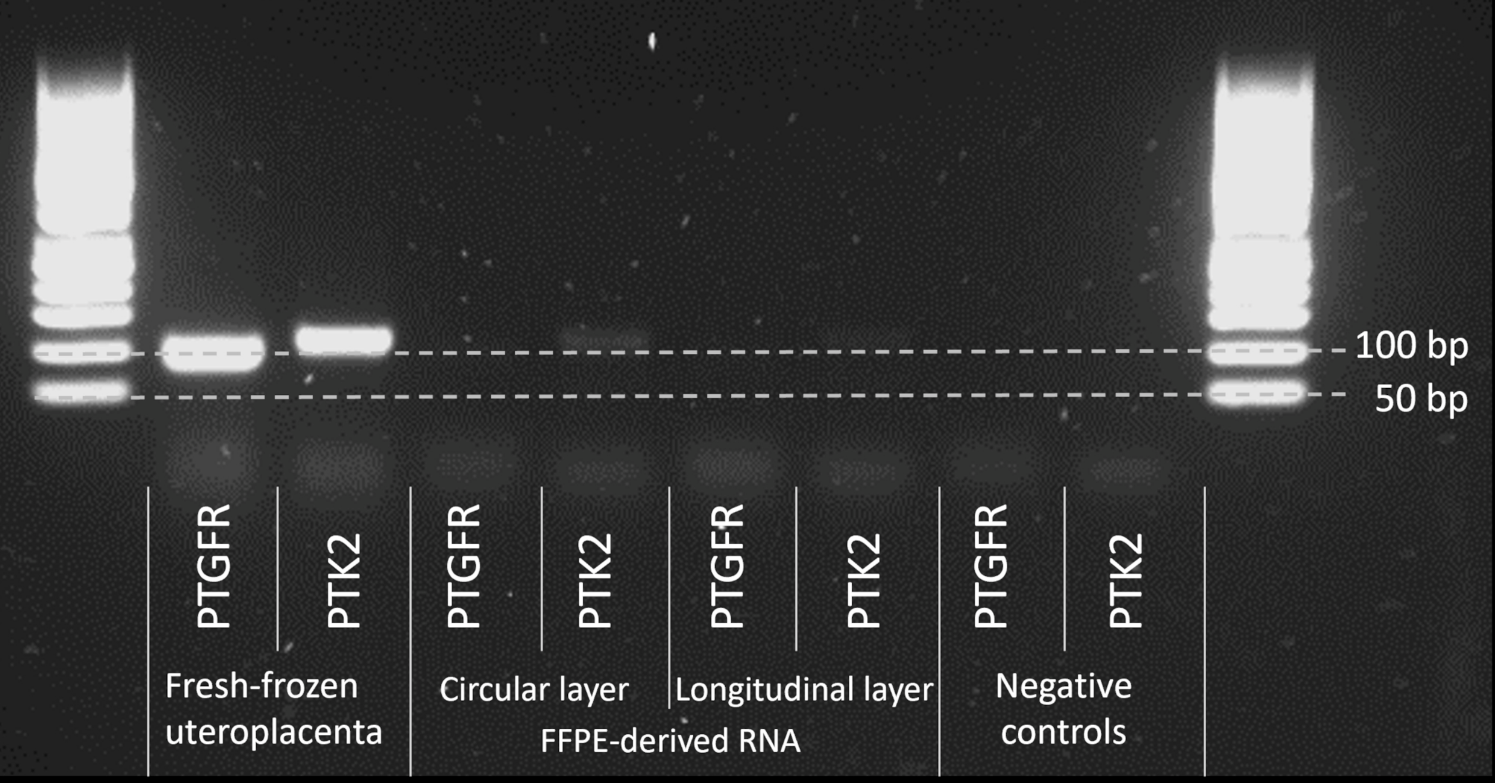


**Figure S1** RT-PCR to determine the mRNA expression of PTGFR and the reference gene PTK2 in fresh-frozen uteroplacental tissue compared to formalin-fixed paraffin-embedded (FFPE) tissue used for separation and individual analysis of myometrial layers. Amplicon length: PTGFR: 91 bp, PTK: 104 bp.
